# Study of the natural course and specific immunity after herpes zoster in patients with rheumatoid arthritis receiving biologic DMARDs

**DOI:** 10.31138/mjr.28.3.164

**Published:** 2017-09-29

**Authors:** Konstantinos Thomas, Petros P. Sfikakis, Dimitrios Boumpas, Kyriaki Boki, Dimitrios Vassilopoulos

**Affiliations:** 1 Joint Rheumatology Program, Clinical Immunology-Rheumatology Unit, 2^nd^ Department of Medicine and Laboratory, National and Kapodistrian University of Athens School of Medicine, Hippokration General Hospital, Athens, Greece,; 2 Joint Rheumatology Program, 1^st^ Propaedeutic Department of Medicine, National and Kapodistrian University of Athens School of Medicine, Laiko General Hospital, Athens, Greece,; 3 Joint Rheumatology Program, 4^th^ Department of Medicine, National and Kapodistrian University of Athens School of Medicine, Attikon General Hospital, Athens, Greece,; 4 Rheumatology Department, Sismanoglio General Hospital, Athens, Greece

**Keywords:** rheumatoid arthritis, varicella zoster virus, reactivation, immunization

## Abstract

Herpes zoster is a common infection especially in elderly persons. It is caused by reactivation of varicella zoster virus (VZV) that has remained dormant within dorsal root ganglia after primary infection. Besides patients with immunodeficiency and malignancies, zoster incidence is also higher in patients with rheumatic diseases compared to the general population. Especially in the group of rheumatoid arthritis (RA) patients being treated with biologics, the risk seems to be steady among regimens with different modes of action (1.6–2.4/100 patients-years). RA patients receiving tumor necrosis factor (TNF) inhibitors are at increased risk during the first year of treatment. The aim of the current research protocol is to evaluate the natural course of herpes zoster and the effect of biologic and conventional synthetic disease modifying anti-rheumatic drugs (DMARDs) on the VZV-specific cell mediated immunity in RA patients after herpes zoster infection. We will – prospectively – include RA patients who develop herpes zoster, while being treated with biologics (anti-TNF, tocilizumab, rituximab, abatacept) and a control group comprised of patients with herpes zoster [RA patients under non-biologic therapies (corticosteroids/csDMARDs) and age- and sex-matched healthy controls). Titers of IgG specific antibodies against VZV glycoprotein gp1 and the percentage and absolute number of VZV-specific activated CD4+ T cells (CD4+CD69+IFN-γ+) at the time of rash onset and during follow-up will be measured by ELISA and flow cytometry respectively. This study will contribute to the better understanding of various aspects of immune response to VZV in the modern treatment era with biologic DMARDs.

## INTRODUCTION

Herpes zoster is a common infection especially in elderly persons. It is caused by reactivation of varicella zoster virus (VZV) that has remained dormant within dorsal root ganglia after primary infection. It is frequently accompanied by significant morbidity that is mainly manifested as chronic neuropathic pain (postherpetic neuralgia). Its incidence ranges between 3–4/1000 patient-years and increases with age. Non-immunized persons carry a 50% risk for herpes zoster until the age of 85, with 3% of them needing hospitalization,^1,2^ whereas more recent studies have shown an increased risk for stroke after zoster.^3,4^

Herpes zoster and its complications are more frequent in patients with attenuated cell-mediated immunity which is mainly maintained by VZV-specific CD4+ T lymphocytes. Zoster infection drives the expansion of specific CD4+ T lymphocyte population and this fact explains the lower incidence of reinfection in the period after the first event.^5^ Besides advanced age, other well-known conditions that compromise cell-mediated immunity and comprise risk factors for herpes zoster are bone marrow or solid organ transplantation, lymphoproliferative diseases, leukemia and human immunodeficiency virus (HIV) infection. Zoster incidence is also higher in patients with rheumatic diseases, compared to the general population. Especially in patients with systemic lupus erythematosus (SLE) and granulomatosis with polyangiitis (GPA), this risk is up to 20 times the risk of general population. Rheumatoid arthritis (RA) patients have a 2- to 5-fold increased risk of zoster infection and hospitalization due to zoster when compared to general population.^6–9^ Risk factors for this group of patients are advanced age, use of corticosteroids, conventional synthetic and biologic disease modifying anti—rheumatic drugs (DMARDs), presence of erosions, large joint involvement, history of joint replacement surgery, malignancies, chronic pulmonary disease, chronic kidney disease and liver disease. Regarding complications, secondary bacterial skin infection, postherpetic neuralgia and Ramsay Hunt syndrome are not more frequent in RA patients. In the group of RA patients being treated with biologics, the risk seems to be steady among agents with different modes of action (1.6–2.4/100 patients-years). RA patients receiving tumor necrosis factor (TNF) inhibitors are at increased risk during the first year of treatment.^10–14^

The role of antigen specific cell-mediated immunity in containing viral reactivation is well established. Specific IFN-γ secreting CD4+ T lymphocytes consist 0.1% of mononuclear blood cells in immunized or naturally infected young adults.15 We already know that cell-mediated immunity starts to wane from the first years of adulthood and that results in a decline of VZV specific CD4+ T lymphocytes with time, while this does not happen with VZV antibodies. These cells show a distinct immunophenotype expressing intracellularly multiple cytokines (TNF-α, IFN-γ, IL2) and differentiation markers (CD127) while they show low expression of anergy markers, such as CTLA-4 and PD-1. The kinetics of specific VZV-specific cell-mediated immunity after an initial herpes zoster episode has not been adequately studied. It is well known that the risk for relapse after a given episode is reduced and this is attributed to an enhancement of cell-mediated immunity that is expressed with an increase in VZV specific CD4+ T lymphocytes.^15^ This increase becomes remarkable between 3 and 6 weeks after the infection, declines gradually until the end of the first year and then remains steady for two more years. During reactivation, this population undergoes a functional shift immunophenotypically expressed by increased IFN-γ production, low expression of CD127 differentiation marker and over-expression of CTLA-4 and PD-1 anergy markers.^16^ This finding is in accordance with findings from reactivation of other chronic latent infections, such as tuberculosis and cytomegalovirus (CMV) infection. In contrast, specific antibodies against VZV show positive correlation with development of more severe disease and postherpetic neuralgia.^3^ The natural course of herpes zoster and the effect of modern therapies in VZV-specific cell mediated immunity in patients with RA has not been adequately studied.

A live attenuated vaccine for prevention of herpes zoster in persons over 60 years old was licensed in Greece recently. The vaccine reduces the risk for herpes zoster by 38–70% depending on age and postherpetic neuralgia by 67%. It is also licensed for individuals with a history of herpes zoster. Studies have shown the positive effect of vaccination on cell-mediated immunity by an increase in the counts and breadth of specific CD4^+^ (but not CD8+) T cells.^17^ By now, the vaccine is contraindicated in immunocompromised patients, including those who receive corticosteroids in doses ≥20 mg prednisone daily and those under biologic therapies.

## AIM OF THE STUDY

To evaluate the natural course of herpes zoster and the effect of biologic and conventional synthetic DMARDs on the VZV-specific cell mediated immunity in RA patients after herpes zoster infection.

## METHODS

This is a prospective study that will include RA patients who develop herpes zoster, while being treated with biologics (anti-TNF, tocilizumab, rituximab, abatacept). Inclusion and exclusion criteria are shown in **Tables 1** and **2**, respectively.

**Table 1.** Inclusion criteria.

**Inclusion criteria**
1. **Age ≥18 years**
**2. Written informed consent**
**3. Diagnosis of rheumatoid arthritis (2010 RA/EULAR criteria))**
**4. Treatment with one of the following biologic agents for ≥3 months:** - Infliximab - Adalimumab - Certolizumab pegol - Golimumab - Etanercept - Tocilizumab - Abatacept - Rituximab
**5. Herpes zoster diagnosis by treating physician, based on clinical criteria**
**6. Zoster duration (from the rash appearance until the first evaluation) ≤3 weeks**

**Table 2.** Exclusion criteria.

**Exclusion criteria**
**1. Age <18 years**
**2. HIV infection**
**3. Hemopoietic stem cell or solid organ transplantation**
**4. Active malignancy**
**5. Prescription of chemotherapy during the last 12 months**
**6. Recurrent herpes zoster** (≥1 incidents at the same or other dermatomal distribution with the current event)

Control groups will be comprised of patients with herpes zoster [RA patients under non-biologic therapies (corticosteroids/csDMARDs) and age- and gender-matched healthy controls].

The following clinical criteria will be needed for herpes zoster diagnosis:

- Unilateral vesicular rash with dermatomal distribution, not exceeding the middle line
- Pain or sensory disorders at the distribution of the rash
- Absence of history of similar rash in the same area (in order to exclude cases of relapsing zosteriform herpes simplex infection)

Data regarding RA disease activity, severity and treatment as well as comorbidities will be recorded. More specifically, the following data will be recorded:

- Age
- Gender
- Comorbidities
- Non-RA therapies
- RA duration
- RA disease activity
- Seropositivity (RF or/and anti-CCP)
- Prior treatment with b- and/or csDMARDs
- Current treatment with b- and/or csDMARDs (date of initiation, dose, date of most recent administration)
- Corticosteroid administration (date of discontinuation or, if currently administered, mean dose during the last 4 weeks)
- Type, dose and date of initiation and discontinuation of antiviral treatment and treatment for neuropathic pain)
- In case of admission, duration of hospitalization

The day of rash onset and its distribution will be recorded. Patients will be given the Zoster Brief Pain Inventory (ZBPI) questionnaire, which is used for evaluation of the magnitude and duration of zoster-related pain. Patients will be evaluated at 3, 6, 12, 24 and 48 months after rash onset. At these time points, blood will be drawn from participants and serum and peripheral blood mononu-clear cells (PBMCs) will be isolated. Serum specimens will be stored at −20°C. PBMCs not processed in the same day, will be stored in liquid nitrogen. After thawing, proper PBMC specimens will be considered only those with cell viability >90%, as this will be tested with tryptan blue stain.

### Laboratory parameters

In each participant, the following will be measured:

1. Titers of IgG specific antibodies against VZV glycoprotein gp1 (ELISA) at the time of rash onset and during follow-up (3, 6, 12, 24 and 48 weeks)
2. The percentage and absolute number of VZV-specific activated CD4^+^ T cells (CD4+CD69^+^IFN-γ^+^) at the time of rash onset and during follow-up (3, 6, 12, 24 and 48 weeks) and their correlation with the type of anti-rheumatic therapy (csDMARDs, TNFi, anti-IL6, anti-CD80, anti-CD20). This T-cell population will be measured with flow cytometry, as previously described in the literature.^18,19^ Specifically, PBMCs will be isolated from whole blood and stimulated with specific VZV antigens (VZV lysate, Serion Immunologics), in the presence of co-stimulatory antibodies (anti-human CD28 and anti-human CD49d antibody). VZV control antigen (Serion Immunologics) will be used as negative control, while staphylococcus aureus enterotoxin B (SEB) will serve as positive control. PBMCs will be incubated in 96-well plates at 37°C in 5% CO_2_ environment for 6 hours. After the first 2 hours, brefeldin A (10 μg/ml) is added, in order to block the vesicular export of intracellular cytokines. PBMCs are collected and red blood cells are lysed with the addition of lysing solution. PBMCs are incubated and stained for surface markers CD4 and CD69. Fixation of cellular proteins with fixation buffer for 20 minutes in the dark and washing of the cells with staining solution follows. Next, the cells are re-suspended in permeabilization buffer solution with saponin 0.1% that makes cellular permeable for intra-cellular staining. This procedure is repeated 3 times totally for 10 minutes each time. PBMCs are stained for intracellular IFN-γ in the dark for 20 minutes and then are washed for one more time with staining solution. Finally, FACS analysis (PARTEC flow cytometry) will be used in order to determine the proportion and absolute number of CD4^+^CD69^+^ T cells that express intracellular IFN-γ (CD4^+^CD69^+^IFN-γ^+^).

Given the fact that VZV-specific CD4+ T lymphocytes can be also detected in general population as an immune residual after chickenpox infection, the lower level of detection for this cell population will be calculated from healthy persons of same age and gender with the study participants, without history of herpes zoster infection (n=5).

### Clinical parameters

The following parameters will be calculated and compared among the study groups with the appropriate statistic methods:

1. The burden of the disease (number of affected dermatomes, cranial nerve involvement)
2. The time until full crusting of the rash
3. The magnitude and duration of acute pain
4. The incidence of postherpetic neuralgia, as was previously defined, with the use of ZBPI
5. The incidence of hospitalizations due to zoster and/or zoster complications (ICD-10: B02.0, B02.1, B02.2, B02.3, B02.7, B02.8, B02.9)

## ANTICIPATED BENEFITS

Herpes zoster is a disease with high incidence and morbidity both in the general population and in patients with rheumatic diseases. Biologic therapies have radically changed the treatment of these diseases, although they carry a higher risk for infections, including herpes zoster.

This study will assist in clarifying issues such as:

- The clinical manifestations and severity of herpes zoster in RA patients receiving biologics in comparison with the general population;
- The magnitude and duration of humoral and cell-mediated immune response after an initial herpes zoster event in RA patients receiving biologics in comparison with the general population;
- The optimal time for immunization of this group of patients after herpes zoster (in case the vaccine will be licensed for this indication);
- The incidence and characteristics of neuropathic pain and its possible correlation with immune response in RA patients receiving biologic therapies.
